# Preclinical Study in Vivo for New Pharmacological Approaches in Inflammatory Bowel Disease: A Systematic Review of Chronic Model of TNBS-Induced Colitis

**DOI:** 10.3390/jcm8101574

**Published:** 2019-10-01

**Authors:** Inês Silva, Rui Pinto, Vanessa Mateus

**Affiliations:** 1 H&TRC–Health and Technology Research Center, ESTeSL–Lisbon School of Health Technology, Instituto Politécnico de Lisboa, 1990-096 Lisbon, Portugal; ines.silva@estesl.ipl.pt; 2 iMed.ULisboa, Faculdade de Farmácia, Universidade de Lisboa, 1990-096 Lisboa, Portugal; rapinto@ff.ulisboa.pt; 3 JCS, Dr. Joaquim Chaves, Laboratório de Análises Clínicas, Miraflores, 1495-069 Algés, Portugal

**Keywords:** inflammatory bowel disease, experimental colitis, chronic models, trinitrobenzene sulfonic acid-induced colitis

## Abstract

The preclinical studies in vivo provide means of characterizing physiologic interactions when our understanding of such processes is insufficient to allow replacement with in vitro systems and play a pivotal role in the development of a novel therapeutic drug cure. Chemically induced colitis models are relatively easy and rapid to develop. The 2,4,6-trinitrobenzenesulfonic acid (TNBS) colitis model is one of the main models in the experimental studies of inflammatory bowel disease (IBD) since inflammation induced by TNBS mimics several features of Crohn’s disease. This review aims to summarize the existing literature and discuss different protocols for the induction of chronic model of TNBS-induced colitis. We searched MEDLINE via Pubmed platform for studies published through December 2018, using MeSH terms (Crohn Disease.kw) OR (Inflammatory Bowel Diseases.kw) OR (Colitis, Ulcerative.kw) AND (trinitrobenzenesulfonic acid.kw) AND (disease models, animal.kw) AND (mice.all). The inclusion criteria were original articles, preclinical studies in vivo using mice, chronic model of colitis, and TNBS as the inducer of colitis and articles published in English. Chronic TNBS-induced colitis is made with multiple TNBS intrarectal administrations in an average dose of 1.2 mg using a volume lower than 150 μL in 50% ethanol. The strains mostly used are Balb/c and C57BL/6 with 5–6 weeks. To characterize the preclinical model the parameters more used include body weight, stool consistency and morbidity, inflammatory biomarkers like interferon (IFN)-γ, myeloperoxidase (MPO), tumor necrosis factor (TNF)-α, interleukin (IL)-6, and IL-10, presence of ulcers, thickness or hyperemia in the colon, and histological evaluation of the inflammation. Experimental chronic colitis is induced by multiple rectal instillations of TNBS increasing doses in ethanol using Balb/c and C57BL/6 mice.

## 1. Introduction

Inflammatory bowel disease (IBD), including Crohn’s disease (CD) and ulcerative colitis (UC), is characterized by chronic and intermittent inflammation of the intestinal mucosa [1]. IBD has been a world health-care problem with its prevalence exceeding 0.5% of the population in westernized countries [2], and growing incidence in newly industrialized countries [3]. The IBD symptoms depend on the intestinal affected segment and usually include diarrhea often with blood, colic abdominal pain, and fecal urgency. Beyond these, other unspecific symptoms may occur like fever, loss of appetite and weight, fatigue, and primary amenorrhea [4].

Although CD and UC include common features, they are distinguished by different pathophysiological aspects and clinical manifestations [1]. CD is featured by transmural and discontinuous inflammation in the entire length of the gastrointestinal tract, but mostly affects the terminal ileum and perianal region [5,6]. Conversely, in UC, the inflammatory lesions are typically confined to the mucosa and affect the large intestine, beginning in the rectum with the eventuality of spreading to rest of the colon [7].

Pathogenesis of IBD is not fully understood but two broad hypotheses have arisen regarding its fundamental nature. The first contends that the primary dysregulation of the mucosal immune system leads to excessive immunologic responses to normal microflora. The second suggests that changes in the composition of gut microflora and/or deranged epithelial barrier function elicit pathologic responses from the normal mucosal immune system. Currently, it is well accepted that IBD is indeed characterized by an abnormal mucosal immune response but that microbial factors and epithelial cell abnormalities can facilitate this response [8]. So, a complex interplay between genetic predisposition, environmental trigger, and an aberrant immune reaction contributes to disease initiation and its progression [9,10]. However, CD and UC present different cellular responses in the context of intestinal inflammation, while CD is a dominantly T helper (Th)1 and Th17 mediated process, UC seems to be a Th2 disorder [11].

In general, currently used medical therapy in IBD consists of salicylates, corticosteroids, immunomodulators, and biological therapy. These drug treatments have the aim to induce and maintain the patient in remission and ameliorate the disease’s secondary effects, rather than modifying or reversing the underlying pathogenic mechanism [12]. Their use may result in severe side effects and complications, such as an increased rate of malignancies or infectious diseases [13]. Drug delivery to the appropriate site(s) along the gastrointestinal tract also has been a major challenge, and second-generation agents have been developed with improved drug delivery, increased efficacy and decreased side effects [14]. Furthermore, IBD patients depend upon continuous medical support, thus contributing to a considerable economic impact. The annual healthcare costs were estimated at around 4.5–5.6 billion euros, in Europe, and 6.3 billion dollars, in the United States of America [2].

For many years, there have been numerous efforts to find a new effective method that would allow controlling specifically unwanted immune responses that occur during the autoimmune reaction [15]. For all these reasons, the development of preclinical studies that allow evaluating other therapeutic alternatives is essential to improve the pharmacological approaches in the treatment of IBD.

Animal models of IBD play a pivotal role in the development of new therapeutic approaches to the treatment of IBD and dissect the possible mechanism of action of a particular drug [6]. Chemically induced models are one of the most commonly used to study IBD, because they are toxic to colonic cells that generate intense inflammatory response and recruitment of inflammatory cells, representing some of the characteristics observed in human disease [16,17]. Since Morris first described it in 1989, 2,4,6-trinitrobenzenesulfonic acid (TNBS)-induced colitis model has been very popular, because a single rectal administration in rats, mice, guinea pigs, dogs, and/or rabbits produce rapid, reliable, and reproducible disease. This animal model, generally accepted as one of the best models for the non-clinical study of new therapy with indication for treating or relieving IBD-associated symptoms, is an efficient method, since it promotes a transmural colitis (Th1-mediated immune response) with severe diarrhea, weight loss, and rectal prolapse, an illness that mimics some characteristics of CD in humans [10,18,19].

Our research group has developed previous preclinical studies in an acute model of TNBS-induced colitis, testing some pharmacological approaches with new drugs, which presented beneficial effects in the progression and treatment of IBD [20,21,22,23,24,25]. However, IBD is a chronic disease and the development of a standardized and validated induction method for chronic colitis is useful to study new metabolic pathways and, consequently, new pharmacological approaches [10,17].

In the literature, there is no consensus about the induction method and several original articles have been published with different ways to induce a chronic model of TNBS-induced colitis, using different doses, frequency of TNBS administrations, strains, gender and ages of mice. By this point of view, this study aims to identify, summarize, compare, and discuss different protocols for the induction of chronic model of TNBS-induced colitis, through PRISMA methodology. This review will allow the summarization of a standardized method of induction for the research of new therapeutic approaches in IBD, contributing to a more effective and safe treatment.

## 2. Material and Methods

### 2.1. Search Strategy

**Figure 1:**
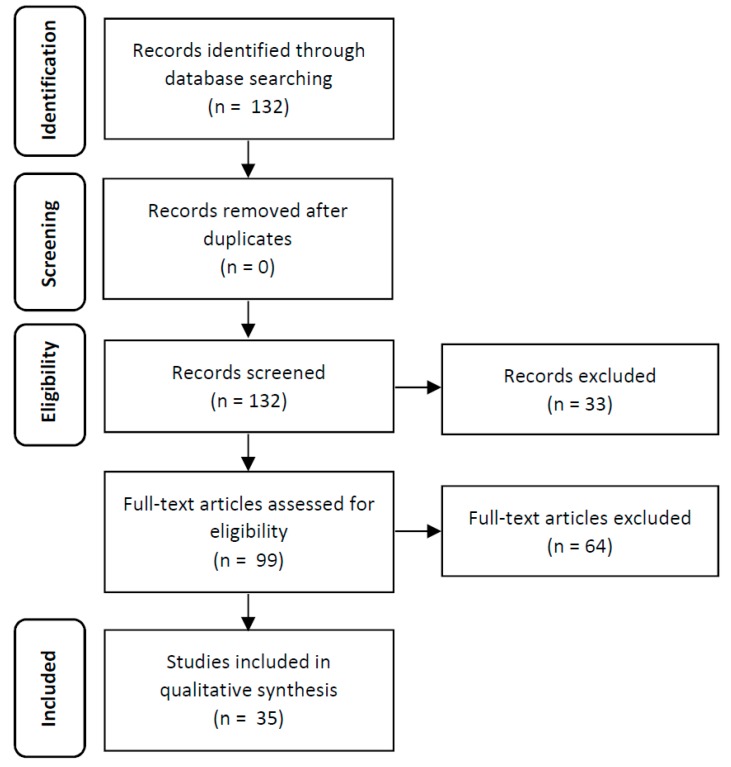
PRISMA flow diagram showing results of the literature search.

Following the establishment of a review protocol based on PRISMA methodology, electronic database MEDLINE via PubMed platform were searched from initiation up to December 2018 for all studies with chronic models of TNBS-induced colitis in mice. The search strategy included inserting the keywords in the MeSH Database to find MeSH terms. Combinations of the keywords were performed. The search expression was: (Crohn Disease.kw) OR (Inflammatory Bowel Diseases.kw) OR (Colitis, Ulcerative.kw) AND (trinitrobenzenesulfonic acid.kw) AND (disease models, animal.kw) AND (mice.all). The results of the literature search are outlined in Figure 1.

### 2.2. Selection of Studies

The resulting articles of our specific search expression were selected according the following inclusion criteria: (1) only original articles; (2) studies where a chronic model of colitis is described; (3) studies where the TNBS is the inducer of colitis; (4) preclinical studies in vivo using mice; and (5) articles were published in English. The exclusion criteria are review articles, short communications, case reports, or expert opinions. Abstracts were eliminated in the initial screening and full texts of the remaining articles were retrieved and reviewed. Disagreements between reviewers were resolved by mutual consensus.

### 2.3. Data Extraction

All data were extracted independently by two reviewers into a Microsoft Excel spreadsheet (Windows 10 edition; Microsoft Corporation, Lisbon, Portugal), with disagreements resolved by consensus. The following information was extracted from each study independently by the two reviewers: TNBS-related parameters (number of administrations, dose, volume, vehicle, and presensitization), mice-related parameters (strain, gender and animal age), model characterization (clinical signs and symptoms, biomarkers, macroscopic evaluation, and histological evaluation), authors and year of publication.

## 3. Results

After application of the search expression, the electronic database identified 132 publications, which were subsequently screened according to the inclusion/exclusion criteria (Figure 1). After abstract analysis, no duplicates were identified; however, 33 original articles were excluded because they were not within the scope of the work. The reasons for the excluded articles were: induction of an acute colitis model (*n* = 20); induction of a chronic inflammatory alveolar bone loss (*n* = 1); preclinical studies using rats (*n* = 6); did not use TNBS as colitis inducer (*n* = 1); the induction method is not described (*n* = 1); the article was written in Chinese (*n* = 1); and the article corresponds to a review (*n* = 3). Of these, 99 published articles appeared to be relevant for the study question and were retrieved for further evaluation. From the remaining ninety-nine identified articles, sixty-four full-text articles were excluded as ineligible, based on the inclusion criteria described previously. The reasons for the excluded articles were induction of an acute colitis model (*n* = 52); preclinical studies using rats (*n* = 2), and the induction method was not described (*n* = 10). Thus, 35 original articles were included in the qualitative analysis, since all of these studies have described a chronic model of TNBS-induced colitis in mice (Table 1).

Animal model studies mimic the pathogenesis of IBD in humans and allow testing of new pharmacological approaches [26]. Clinically, there are several types of animal models of IBD, which can be divided based on how the disease is produced: Those that express intestinal inflammation spontaneously (spontaneous models), those in which intestinal inflammation can be induced by specific immunological (immunological models) or chemical agents (chemically induced models), those that are genetically engineered by gene knockout, knockin, or transgenic methods (genetically engineered models), and the last includes adaptative transfer models (adoptive transfer models) [6,10,27].

The chemically induced models to study IBD are the models studied in greatest detail so far. Among the various chemically induced colitis models, dextran sulfate sodium (DSS)-induced colitis and TNBS-induced colitis models are the most widely used to induce IBD [6,28]. These colitis models are appropriated to developing and testing novel therapeutic strategies for the treatment of IBD. Moreover, the plausible mechanism of action of a particular drug can be illustrated using a suitable colitis model [6]. Differences between models may reflect the different subgroups of patients with IBD [29]. The scientific evidence suggests that TNBS-induced colitis promotes a Th1 response, resembling CD in humans [30]. In turn, the DSS-induced colitis model promotes a Th2 response, resembling UC in humans [6]. In practice, this pattern of T-cell differentiation is associated with distinct functional activities: Th1 cells are the key players in delayed-type hypersensitivity reactions, whereas Th2 T cells are potent inducers of antibody-mediated immunologic reactions [31]. Additionally, Antoniou et al. (2016) describe that TNBS-induced colitis includes the development of a transmural inflammation that closely resembles histopathological lesions that develop in human CD [32].

DSS-induced colitis is a reproducible model that morphologically and symptomatically resembles UC in humans [17,33]. The commonly used protocol for DSS-induced colitis in mice is to add DSS to drinking water [33,34,35] in a dose range of 2%–10%, by repeated exposure administering in three to five cycles, punctuated with recovery periods [10,33]. The severity of DSS-induced colitis model depends the dose, duration of administration and animal strain (C3H/HeJ and Balb/c mice strains are more susceptible [33,34,35]. Another factor that can influence the severity and susceptibility of exposure to DSS is gender (males are more susceptible). However, this model has the disadvantage of being very expensive.

The colitis model with TNBS is one of the most widely used chemically induced models of IBD, as it was an easy induction, rapid, reliable, robust, and a highly reproducible animal model of intestinal inflammation [10,26,29,36,37]. The induction of the disease occurs quickly and appears 4 to 7 days after intrarectal administration of the TNBS, promoting acute or chronic colitis depending on the dose and frequency of administration [10,17,38].

**Figure 2:**
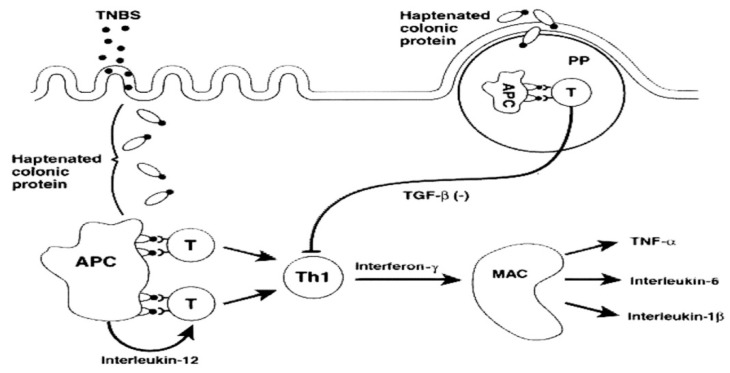
Mechanism of action of 2,4,6-trinitrobenzenesulfonic acid (TNBS). Inducing experimental colitis [40].

Experimental colitis is induced by rectal instillation of TNBS in ethanol, which will produce a reaction with certain groups of amino acids in the intestinal mucosa and colonic bacterial proteins, making them immunogenic through a process called haptenation [10,36,38]. TNBS haptenates autologous colonic proteins with a trinitrophenyl (TNP) moiety and induces an IL-12 mediated Th1 T cell transmural colitis (Figure 2), which resembles human IBD, both on a histologic and immunologic level [10,39].

The use of reagents other than trinitro or dinitro hapten moieties may also result in altered results in terms of the degree of inflammation or mortality rate. It should be noted that substances such as methyl sulfonic acid can also bring about macroscopic inflammation. However, this is not accompanied by any chronic length of illness and does not give rise to a cross-reactive immunologic response [38].

Protocols of the chronic TNBS-induced colitis model are not standardized, such as the dose of TNBS, the depth of TNBS administration, the animal strain, and the time point for model evaluation [10,27]. Since so many animal models are now available, investigators must take into consideration all these variables for application in the preclinical testing [36]. Thus, we will continue to compare and discuss some important parameters for this animal model of colitis, such as the TNBS-related parameters, the mice-related parameters, and the model characterization.

### 3.1. TNBS-Related Parameters

#### 3.1.1. Number of TNBS Administrations

Most studies consider that the chronic model of colitis can be induced by more than one administrations (*n* = 20). Analyzing Table 1, chronic colitis was induced with schemes of two (*n* = 8), three (*n* = 4), or four or more administrations of TNBS (*n* = 8). Tomasello et al. (2015) defend that repeated administrations of TNBS are preferred, resulting in a local Th1 response that has the characteristics of Crohn’s disease. Moreover, these authors consider that TNBS-induced colitis by seven weekly intrarectal administrations of TNBS most likely reflects the chronic phase of Crohn’s disease [76]. According to Wirtz et al. (2017), a dose-escalating strategy demonstrated to be more successful for the induction of chronic colitis [17]. In the same perspective, Yang et al. (2012) defend that recurrent colitis model can be induced by instilling TNBS into the colon through a cannula, but is followed by a second instillation with a lower dose of TNBS into the colon 14 days after the first induction of colitis [77]. Also, Elson et al. (1996) defends that recurrent colitis can be achieved by repeated enemas of TNBS, but never by oral feeding of TNBS, since this will indorse significant oral tolerance [63].

Based on the expertise of our research group in the development and validation of an acute model of TNBS-induced colitis, all parameters under evaluation corroborated that the damage became maximal at day 4 after the induction with a single administration of TNBS. After day 4, mice progress to a chronic phase of the disease, showing the same symptoms but more lightly [20,21,22,23,24,25].

Indeed, a single administration of TNBS can induce a chronic model; however, the collected samples (blood or colonic tissue) have to be analyzed after day 6 from induction. Multiple TNBS administrations strategy may be another different approach to achieve a chronic model, using the same dose or with escalating doses.

The disease severity and the time required to produce the injury may vary between laboratories. Concretely, TNBS presents an infiltration of inflammatory cells within 2h after administration, but typical signs of chronic inflammation can develop after 48h [78]. Therefore, the acute transmural damage became maximal from 3 days to 1 week to after instillation (acute model) and resolved within 2 weeks (chronic model). However, if multiple TNBS administrations are used, the colonic inflammation can gradually progress lasting for about 8 weeks [19,27,79].

Additionally, it is important to refer that in models involving several administrations, we must take into account that TNBS hapten reagent should not be used for experimental purposes if more than 3 months have elapsed. The disease severity and clinical course may be altered with suboptimal reagent [38].

#### 3.1.2. Dose

In the analyzed studies, the TNBS dose ranges between 0.3–5.0 mg per mouse. The average dose used was 1.2 mg and the mode was 1.4 mg of TNBS. However, 77% of the articles analyzed use a dose higher than 0.5 mg. It is important to note that the highest dose used was 5.0 mg in C57BL/6 strain and 3.0 mg in Balb/c mice.

According to Scheiffele & Fuss (2001), adjusting the respective doses of TNBS may bring about a spectrum of disease, from acute (0.5 mg/mouse) to chronic (3.75 mg/mouse). Higher dosages of TNBS (>3.75 mg) may lead to more acute mortality rate due to massive colitis, necrosis, and colon perforation, while lower dosage, (<0.5 mg) may cause short-lasting, weak or even completely absent colitis activity [10,17,18,38]. Tomasello et al. (2015) report that a dose of 50 mg/kg of TNBS, which corresponds to 1 mg/mouse, produces colonic inflammation and ulcers avoiding the risk of massive loss of animals [76]. It is important to note that individual optimization of administering TNBS concentrations is essential, particularly to generate chronic colitis [38,76].

In fact, the optimal TNBS dose to induce colitis fluctuates due to several key factors, including genetic background, gender, age, body weight, as well as sterility conditions of the animal facility, and will have to be determined individually [36,38,39]. Engel et al. (2011) report that higher concentrations of TNBS may be used in the C57BL/6 strain, because this strain is less susceptible to TNBS-induced colitis. Thus, higher TNBS concentrations are necessary to induce colitis for several weeks [80].

Regarding sterility conditions of the animal facilities, germfree conditions and a clean environment have a large impact on disease outcome since the induction may vary within a given facility over time, depending on the sanitation [17,38]. In this sense, all diagnostic studies should be performed in a designated area exclusively for the colitis induction with TNBS. The cohabitation of the experimental mice with other strains of mice or pathogens may alter the immune response and the induction of colitis dramatically [38].

#### 3.1.3. Volume

Before administering any substance to an animal, the investigator should make appropriate decisions not only about the dose to be administered but also about the volume. The injection volume is limited by any toxicity of the substance and by the size of the mouse. Excess volumes of solution can startle the animal. In this sense, the maximum recommended volumes are shown in many guidelines according to the route of administration. Rectal administration is an enteral administration made directly in the gastrointestinal tract that can be performed using soft small-gauge flexible tubing with a dosing syringe attached to the end. In the mouse, the injection volume limit on rectal administration is 500 µL [81].

In this review, all studies use between 50–500 μL of injected volume, which is in agreement with the literature. However, in most cases, authors opted to administrate lower than 150 μL (*n* = 20) and some did not even mention the injected volume (*n* = 12). Only in one study, 500 μL of the injected volume was used.

According to our experience with rectal administration in mice, the risk of leakage is higher for volumes above 100 μL. Although the literature does not recommend an ideal volume for rectal administration in mice, some variables have to take into account. Regardless of whether the mice have fasted, there is always the possibility of the presence of feces in the colon that limits the space for TNBS administration of a given volume. Even with very small volumes, the possibility of reflux is high, due to several situations such as lack of practice in the technique, anatomical positioning of the descending colon, and injection rate of the volume to be administered. For this reason, the Trendelenburg position is usually adopted in this protocol after TNBS administration. This procedure is a variation of the supine position where the upper back is lowered, and the lower limbs are raised during the following few minutes after administration to avoid the rectal reflux of TNBS. Therefore, it is preferable to administer the desired dose of TNBS in a reduced volume to ensure that the entire solution is retained in the colon because if there is reflux, even if reduced, it compromises the correct validation of the model.

#### 3.1.4. Vehicle

Chemically induced models require the co-administration of a substance that temporally disrupts the mucosal integrity and allows the colitogenic components to access the mucosal immune system [82].

The range of ethanol concentrations used in the analyzed articles was between 10%–80%. However, most studies use ethanol between 45% and 55% (*n* = 27) as a TNBS vehicle. In the literature, the dosage of ethanol is considered optimal ranging between 30% to 50% [36,38], which is by following our review. On varying alcohol concentration, it was found that the pathological score, such as inflammation and visceral hyperalgesia, was more significant in TNBS 50% ethanol-treated animals [27]. So, the ethanol at 50% is the most advised to disrupt the intestinal barrier and enable the translocation of the TNBS into the submucosal layer.

Some authors use lower concentrations of ethanol because they want to avoid ethanol interference in inducing damage to the intestinal epithelium. Nevertheless, studies using ethanol at 50% as TNBS vehicle in colitis model already proved that ethanol does not affect in the observed colon lesion [67,70,71,72,75]. In our colitis model, the findings corroborate the same [20,21,22,23,24,25].

The use of ethanol is only required to break the intestinal barrier, increasing its permeability [10,17,36,37,83]. Ethanol permeabilizes the epithelial layer that separates the luminal contents of the colon from the cells of the mucosal immune system, allowing the penetration of TNBS in the bowel wall [26,83].

#### 3.1.5. Presensitization

The presensitization procedure consists in a subcutaneous TNBS administration before the induction protocol, which helps to produce a specific Th1 response upon reapplication of TNBS by a mechanism of type IV hypersensitivity reaction [37]. Compared to the rectal administration of TNBS, the skin contact reaction with this chemical induces delayed and not persistent hypersensitivity. This is probably due to TNBS effector immune cells, which cross-react with ubiquitous mucosal antigens and thus continues to be stimulated even after the proteins that have been secreted with TNP have disappeared [38].

Most of the analyzed articles do not use presensitization. The presensitization was only applied in six studies. Nevertheless, they did not present a concrete improvement from this procedure. Indeed, the literature reports that this previous procedure brings benefits for the resulting induction, allowing the alleviation of disease severity and reducing the mortality rates associated with the TNBS administration [15]. The advanced hypothesis is that the excessive inflammatory response after the first rectal administration of TNBS decreases through the production of TNBS antigens. However, the presensitization is not usually used by the authors and there is no proof of its benefits.

### 3.2. Mice-Related Parameters

#### 3.2.1. Strain

Preclinical studies of experimental colitis have been developed in rats, mice, pigs, rabbits, nonhuman primates, and dogs [84,85]. Nevertheless, the use of mouse models allows the use of larger cohort sizes, decreases the number of therapeutic reagents required, and also enables the incorporation of animals with specific genetic modifications [86]. Therefore, the susceptibility to TNBS-induced colitis varies significantly between mice strains [10,36,83]. If susceptible strains of mice are used, over 90% should develop TNBS-induced colitis [38].

According to the Table 1, the most frequently used strains to induce chronic colitis were Balb/c (*n* = 21) and C57BL/6 mice (*n* = 10) [17,38,83]. The SJL/J mice (*n* = 4) strain is not usually used for the TNBS-induced colitis.

Curiously, TNBS-induced colitis was initially described in SJL/J mice, which was considered a mouse strain with high susceptibility for the induction of colitis [19] and, currently, this fact remains well accepted in the scientific literature [17,83]. So, these findings seem to propose that the authors are not using the most appropriate strain to induce chronic colitis.

But nowadays, various other mouse strains are also frequently used for the development of colitis, such as BALB/C and C57BL/6 [6]. Compared to SJL/J and Balb/c mice, C57BL/6 mice are relatively resistant to TNBS-induced colitis [10,17]. However, when using presensitization, this strain develops significant TNBS colitis [10].

Even so, this was not demonstrated in our review, because the analyzed studies with presensitization have used the Balb/c strain. The use of presensitization in Balb/c mice may be related to the fact that this strain is less susceptible to TNBS-induced colitis than the SJL/J strain, or to reduce the mortality rate associated with the TNBS administration. It is important to consider that the variability among mice strains require also the optimization of the TNBS concentration [36], as we have mentioned before.

#### 3.2.2. Gender

Analyzing the selected studies, the findings allow concluding that here is no trend towards gender. The number of articles referring to the use of males or females is similar. Some articles do not even refer to gender (*n* = 10) and, for two of them, male and female are used in the same study. However, the literature is not consensual in the matter. On the one hand, some authors refer that although both males and females can develop TNBS-induced colitis with the same clinical characteristics, males can develop more expressive and chronic disease [38]. On the other hand, some authors consider that IBD does not have an associated hormonal component, so it makes sense that there are no differences between genders [18]. So, this factor remains unclear, however, the results seem did not change with the gender.

#### 3.2.3. Age

Relatively to animal age, the analyzed articles are using mice with a large range of ages, namely between 4 to 16 weeks. There are studies with mice up to 8 weeks (*n* = 16), mice older than 8 weeks (*n* = 15); and some studies do not even mention the animal age (*n* = 4). However, according to Scheiffele and Fuss (2001), colitis induction should be performed at 5 to 6 weeks of age, because younger animals have a greater success rate of induction. Additionally, animals up to 4 weeks of age suffer an increased mortality rate, probably because of the TNBS induced toxicity. In contrast, animals over 8 weeks of age have a diminished sensitivity [38]. Although this could be overpass with a slight increase of the TNBS dose to allow the induction of intestinal inflammation, in fact, there is no evidence about its impact on the success of the induction and the mice morbidity slight increase in the dose.

Some articles define the weight instead of the weeks. In this case, the used average weight is around 20g (data not shown), which conforms to the weight of an adult mouse [81]. Regarding this parameter, it is being considered that colitis induction should be performed in animals between 18–20g of body weight, what is by following our results and directly related to the previous explanation.

### 3.3. Characterization of the Preclinical Model

The evaluated parameters are transversal to all analyzed articles. In general, authors evaluate clinical signs and symptoms, biochemical markers, and observed macroscopic lesions, and then make a histological assessment of the colon samples. However, it is important to note that not all articles characterize its preclinical models by the same parameters. Below, we briefly describe each one in terms of the purpose of the analysis and the type of sample used. The main objective was to identify the period in which chronic intestinal lesion pattern would have been reached by the induction method used in the present study.

#### 3.3.1. Clinical Signs and Symptoms

In the TNBS-induced colitis model, clinical signs and symptoms usually include the monitoring of body weight, morbidity, stool consistency/diarrhea, and anus appearance. These parameters should be observed daily during the experimental period. According to most analyzed articles, the TNBS-induced colitis promotes body weight loss (*n* = 25) and soft stools or diarrhea (*n* = 9), accompanied by a general deterioration in their appearance (*n* = 4). These are in agreement with other authors who describe that the animals develop visible signs of disease, with piloerection and decrease of the activity level [26,38]. The clinical manifestations of chronic colitis typically peak by 2 weeks and can be followed by partial recovery or death [19,50,63].

Although the clinical signs and symptoms appear to be less sensitive parameters, almost all observed studies with this preclinical model have shown the necessity of monitoring them (*n* = 28). Normally, weight loss is progressive, with 10% observed in the first 1 to 2 days, culminating at 20% to 30% by 7 days [38]. Mortality typically increases with weight loss. Expected mortality remains about 20% to 25% through 7 days [38]. Regarding the stool consistency, mice present throughout the study alterations of intestinal motility characterized by diarrhea or soft stools and severe edema of the anus, consistent with other research groups [18,87].

#### 3.3.2. Biochemical Markers

A biomarker concentration can be detected or measured in blood or tissues, reflecting the severity or presence of pathology. The determination of biochemical markers allows a precise quantification, which is used in almost all studies (*n* = 25). The majority of articles analyzed include serum collect blood samples to analyze biochemical markers used to determine the severity of colitis [88,89,90].

The biomarkers described in the analyzed articles can be divided into inflammatory factors: interferon (IFN)-γ (*n* = 13), tumor necrosis factor (TNF)-α (*n* = 11), myeloperoxidase (MPO) (*n* = 11), interleukin (IL)-6 (*n* = 11), IL-12 (*n* = 5), IL-1β (*n* = 5), IL-10 (*n* = 7), and immune response cells: CD4^+^ lymphocytes (*n* = 6).

The pro-inflammatory cytokines, IFN-γ, TNF-α, IL-6, IL-12, IL-1β, and the anti-inflammatory cytokine, IL-10, should be measured in the colon. These biomarkers confirm that the use of TNBS-induced colitis model promotes a significant increase in the levels of these pro-inflammatory cytokines in the colon. Cytokines are critical in immune responses, IFN-γ, TNF-α, IL-6, IL-12, and IL-1β are released after triggering the inflammatory process. The increased values of this pro-inflammatory cytokines are related to IBD pathogenesis, once they are augmented in colitic tissue [91,92]. On the other hand, the presence of anti-inflammatory cytokines, such as IL-10, suggests a decreased serum value, in different data, consistent with the expected inflammatory process instigated by TNBS induced colitis [93,94].

The concentration of MPO represents a marker of neutrophil infiltration in the tissue and indirectly allows the quantification of neutrophil accumulation through a colon sample. In the presence of inflammation, the MPO enzyme is released from the colonic mucosa, allowing direct correlation of its release with the values in the systemic circulation [95]. Different data are consistently affirming that TNBS induced colitis is characterized by an increase of MPO activity, suggesting exacerbation of the colon inflammation. On the other hand, the control groups presented a residual MPO activity, compatible with the absence of an inflammatory process [15].

Concerning to CD4^+^ lymphocytes, they are an essential part of the human immune system, and it is very well reported that administration of TNBS is associated with predominant activation of Th1 mediated immune response [96]. This response manifests by dense infiltration of local CD4^+^ T cells [6] in the experimental groups compared with groups without TNBS administrations.

Although none of the articles analyzed refer to fecal hemoglobin, our research group used fecal hemoglobin, an extremely sensitive parameter, in the acute colitis model. A quantitative method by immunoturbidimetry is used to evaluate fecal hemoglobin, as an index of hemorrhagic focus. The determination of fecal hemoglobin allows the diagnosis and evaluation of various colorectal diseases, once it determines the intensity of the hemorrhagic focus in the damage of colonic tissue [97,98,99]. In this sense, we expect to have high values of fecal hemoglobin in the TNBS groups as opposed to the control group, where we suppose that they present residual fecal hemoglobin concentrations.

Urea, creatinine, and alanine aminotransferase (ALT) are also biomarkers not evaluated by the articles analyzed in this review. However, based on the expertise of our research group in the development of an acute model of TNBS-induced colitis, these parameters allow evaluating the extra-intestinal influence of our induction model. These biochemical markers, nonrelated directly with the intestine, are representative of external and consequent manifestations of the inflammation with TNBS. Urea and creatinine are determined as markers of renal function, and ALT is determined to be a marker of hepatic function. These biochemical markers are evaluated spectrophotometrically. Consistent with the literature, the higher serum levels represent extraintestinal manifestations and secondary effects involved with almost every organ system and some of the most frequently involved organs are the liver and kidney [100]. TNBS-induced colitis is therefore expected to show a significant change in renal and hepatic functions compared to the control groups, characterized by increased levels of these markers in serum. According to our data, most of the biomarkers evaluated to study IBD are specific inflammation markers (IFN-γ, TNF-α, IL-6, IL-10, and MPO) that intend to represent and prove the reliability of the induction method used, as well as to verify the occurrence of inflammation in the intestine.

#### 3.3.3. Macroscopic Evaluation

Macroscopically, several parameters can be analyzed in the necropsied colon, such as weight and length of the colon, wall thickness, hyperemia, ulceration, and adhesions, as described by Morris et al. (1989). These parameters are consistent in most of our data (*n* = 23) as a reflection of its importance in the evidence of the disease.

Colon length (*n* = 12) is measured as a marker of tissue integrity, determined using a measuring scale and analyzed for intestinal damage. Findings from other research groups point to colonic shortening after TNBS treatment, demonstrating TNBS-induced colitis promotes a reduction in the colon length [15,101].

Relatively to the bowel thickness (*n* = 9), it is expected an increase in the thickness of the gut wall in the presence of inflammation. Maximal thickness, correlated to colon cyclooxygenase (COX-2) expression, is significantly increased in TNBS groups compared to the control groups [102]. COX-2 is an inflammatory marker involved in angiogenesis processes and chronic inflammation and be more relevant in chronic colitis assessment [103].

The macroscopic observation of the bowel also demonstrates ulcerations (*n* = 8) and hyperemia (*n* = 8). The analyzed articles describe marked hyperemia and ulcers in the colons from mice in the experimental groups, whereas the colons from the mice in control groups showed no or only slight inflammation. Both parameters are consistent with the presence of IBD [19,32].

#### 3.3.4. Histological Evaluation

Histopathology analysis is based on the colon samples, removed shortly after sacrifice, fixed in an appropriate solvent, and then processed. The histologic analysis method allows a qualitative evaluation of the analyzed sample. However, there are several scores based on different parameters to characterize quantitatively the colon lesions induced by TNBS [50,57,61,65,68,70].

In the analyzed articles, the histopathological score of lesions includes the presence of inflammation, the percentage of intestine affected, and the severity of the mucosal epithelial lesion. The colitis severity is evaluated by summing the individual scores, promoting a final colitis evaluation.

The histological evaluation is often used to understand the impact of our study in the colon tissue, beyond the previously observed changes in the other parameters. In our review, almost all studies evaluated the colonic histological changes (*n* = 29). Due to its ability to induce a change in intestinal permeability, promoting damage to the colon tissue and triggering severe inflammation in the intestine, TNBS is a strong cause of histopathological variations in colitis tissues. Concerning the histopathological analysis of the colon, the morphological characteristics in TNBS-induced colitis consistent with a correct experimental induction are generally diffuse transmural necrosis with severe hemorrhage, involving the mucosa, submucosa, muscular and serous layers, and frequently associated with peritonitis [18,92,104,105].

## 4. Discussion

The animal models in IBD have been developed and have contributed greatly to important advances in our current understanding of the immunological, pathological, and physiological features of chronic intestinal inflammation [30]. These findings may provide insight into these potential therapeutic approaches to ameliorate the inflammation and to minimize the morbidity and mortality associated with IBD. However, variability in the results in preclinical studies, due to several conditions such as type of induction method, administered doses and treatment period, difficult the translation of the data for the clinical practice. Careful attention may be required to translate animal studies to clinical settings by ensuring that both safety and efficacy can be modeled [28]. Robust clinical trials providing such potential benefits will be required before the use of these drugs for the management of IBD can be recommended.

In the chronic TNBS-induced colitis model, colonic inflammation is induced in susceptible strains of mice, by intrarectal administrations of the haptenating substance, TNBS, in increasing doses, together with ethanol. The advantage of chronic models compared to acute models is that the latter may provide only limited information about the pathogenesis of human IBDs, as the chemical injury to the epithelial barrier leading to self-limiting inflammation rather than to chronic disease [17].

This systematic review concludes that the chronic TNBS-induced colitis model can be obtained with multiple TNBS administrations, with an average dose of 1.2 mg/mouse (with 20 mg of body weight), in a volume between 100 μL to 150 μL, in 50% ethanol. The strains mostly used are Balb/c and C57 BL/6 with 5–6 weeks with males or females. The most used parameters to characterize this preclinical model include: clinical signs and symptoms (body weight, stool consistency, and morbidity), concentration of inflammatory biomarkers (IFN-γ, MPO, TNF-α, IL-6, and IL-10), macroscopic evaluation of the colon (ulcers, thickness, and hyperemia) and histological evaluation of the colon.

In preclinical studies developed by our research group, we used 100 μL of 2.5% TNBS in 50% ethanol to induce an acute TNBS-induced colitis model. We observed a reduction of the inflammation associated with IBD after administration of erythropoietin, thiadiazolidinone-8, or hemin [20,21,22]. Now, this systematic review allows us to better understand the different methods to induce a chronic TNBS-induced colitis model. Our research group will try to evaluate if the efficacy and safety of these drugs are similar to comparing acute and chronic conditions.

Some other interesting and promising examples of conceptually new strategies in IBD treatment include the fortification of the impaired epithelial barrier, stimulation of innate immune processes via Toll-like receptor (TLR) agonists, or the modulation of the intestinal flora in IBD [106]. It will be interesting to investigate if these new drugs can eventually produce an effect in one of these mechanisms. Future studies are also needed to clarify the relevance of some changes in the drug delivery system could promote in the therapeutic effect since topical application by rectal administration is eventually a good strategy to achieve a more effective and selective treatment with less adverse reactions.

## Figures and Tables

**Table 1:** Outcomes with chronic models of TNBS-induced colitis.

TNBS-Related Parameters					Mice-Related Parameters			Characterization of the Preclinical Model	Reference
Number of Administrations	Dose (mg/mouse*)	Volume (μL)	Vehicle (%Ethanol)	Pre-Sensitization	Strain	Gender	Age (Week)		
(WEEKLY)									
1	2.8	100	_	No	Balb/c, SJL/J, Transgenic	Female	8–12	CSS; BM; M; H	[41]
	2.2		30		Balb/c	Male	_	BM; M; H	[42]
	2.5	-	50		C57BL/6	Female	12	H	[43]
	0.3				Balb/c, SJL/J, C3H/HeJ	_	8–16	CSS; BM	[44]
	2.4	40			C57BL/6	Male	5	CSS; BM; M; H	[45]
	1.7	100			Bagg Albino/c		7	CSS; M; H	[46]
	1.1				Swiis	Female	8–10	CSS; BM; M; H	[47]
					Balb/c		5		[48]
	0.3				C57BL/6		8–10		[49]
					Balb/c, SJL/J		8–16		[50]
	1.7					_	6–8		[51]
	0.3	150			Balb/c		8–10		[52]
	2.0	300			C57BL/6	Male	9–12	BM; M; H	[53]
	2.8	_	80		Balb/c	Female	7	CSS; M	[54]
	0.3	100	50	Yes			6–8	CSS; BM; M; H	[55]
2	1.1–1.1	_	40	No	Transgenic	_	8–16	CSS; H	[56]
	0.3–1.1				Balb/c		_	CSS; BM; H	[57]
	5.0–2.0 or 3.0–2.0	100			C57BL/6, Balb/c	Male		CSS; BM; M	[58]
	0.7	_	50		Balb/c	_	10–16	CSS; BM; H	[59]
	0.8–0.8	100			C57BL/6	Male	6–8	BM; M; H	[60]
	1.7–0.9	120			Balb/c			CSS; BM; H	[61]
	1.4–1.4	150			C57BL/6	_			[62]
	1.1–0.6	500		Yes	C57BL/6, Balb/c, Transgenic	Male, Female	8–16		[63]
3	1.1–1.1–1.1	100	10	No	Balb/c	_	_	CSS; BM; M; H	[64]
	0.5–0.7–1.6	_	50		Balb/c ByJ	Female	7–8	BM; M; H	[65]
	0.4–0.9–1.2		48	Yes	Balb/c	Male	8–10	CSS; BM; H	[66]
	0.4–0.6–1.4		50		Balb/c	Female	8	CSS; BM; M; H	[67]
4 or more	0.3–0.3–0.4–0.4–0.6–0.6	50	30	No	Wild-type and Trangenic	_	8–9	H	[68]
	0.9–0.9–1.1–1.1–1.4–1.4–1.4	100	45		Balb/c	Male	4–5	CSS; M	[69]
	0.6–0.6–1.1–1.1–2.2–2.2	50–100	50				6	CSS	[70]
	0.9 to 1.4 or 2.2 to 3.4	100			Balb/c and C57BL/6	Male, Female	6–8	CSS; M; H	[71]
	1–1–2–2–3–3	200			Balb/c	Male	6	BM; M; H	[72]
	1.1–1.4–1.4–1.4	_	55		C57BL/6		6	CSS; BM; M	[73]
	0.3–0.3–0.4–0.4–0.6–0.7–0.7–0.7		45	Yes	CD-1 outbred	Female	12	CSS; M; H	[74]
	0.6–0.6–0.6–0.6–1.4–1.4–1.4–1.4		50		Transgenic	_	6–12	CSS; BM; H	[75]

**Legend:** CSS: Clinical signs and symptoms (e.g., body weight, mortality, morbidity, stool consistency); BM: Biochemical markers (e.g., TNF-α, TGF-β, IL-6,10,12; IL-1β, IFN-γ, MPO, CD4^+^ lymphocytes); M: Macroscopic evaluation (e.g., ulcers, thickness, hyperemia, colon weight and length); H: Histological evaluation (e.g., inflammation). * mg/mouse: each mouse weighs 20 mg.
